# Reliable estimation of externally validated prediction errors for QSAR models

**DOI:** 10.1186/1758-2946-5-S1-P33

**Published:** 2013-03-22

**Authors:** Désirée Baumann, Knut Baumann

**Affiliations:** 1 Institut für Medizinische und Pharmazeutische Chemie, Technische Universität Braunschweig, Beethovenstraße 55,D-38106 Braunschweig, Germany

## 

In most cases of QSAR modelling the final model used to make predictions, is not known *a priori *but has to be selected in a data driven fashion (e.g. selection of principal components, variable selection, selection of the best mathematical modelling technique). Reliable estimation of externally validated prediction errors under this model uncertainty is still a challenge in chemoinformatics. To fulfil the standards of external validation, the test data set has to be independent not only from model building but also from model selection.

There still is a controversy in the literature how the independent test data set should be chosen and how large it should be. For setting aside a test data set there are basically two different options: 1) a single test data set is set aside and 2) the test data are generated by repeatedly partitioning the available data into test and training set partitions - i.e. cross-validation. Since cross-validation uses the data more efficiently, it is to be preferred in particular for small data sets.

The aforementioned cross-validation step must not be confused with a cross-validation step that might be necessary to select the model! If model selection is also done by cross-validation two loops of cross-validation are necessary [1]. In the inner loop, cross-validation is employed for model selection [2] (also referred to as internal validation) while in the outer loop of cross-validation different test data sets are generated repeatedly that are used to assess the readily selected models (external validation).

In this contribution double cross-validation is evaluated for its ability to estimate prediction errors under model uncertainty. Depending on how double cross-validation is parameterized (test set size, number of repetitions), it either yields biased or highly variable estimates of the prediction error. The sources of bias and variability will be highlighted and recommendations are provided how to determine the test set size in order to obtain a favourable bias-variability trade-off.
